# Clinical and Patient-Reported Outcomes in Non-English-Speaking Patients Undergoing Primary Total Knee Arthroplasty

**DOI:** 10.7759/cureus.116486

**Published:** 2026-09-18

**Authors:** Faith I Wheeler, Justin Turcotte, Jane C Brennan, Saavan Boghara, Paul J King, Andrea H Johnson

**Affiliations:** 1 Orthopedic Research, Luminis Health Anne Arundel Medical Center, Annapolis, USA; 2 Orthopedic and Surgical Research, Luminis Health Anne Arundel Medical Center, Annapolis, USA; 3 Orthopedic Surgery, Luminis Health Anne Arundel Medical Center, Annapolis, USA; 4 Orthopedics, Luminis Health Anne Arundel Medical Center, Annapolis, USA

**Keywords:** clinical outcomes, disparities, non-english speakers, patient reported outcomes, primary total knee arthroplasty

## Abstract

Introduction

Addressing disparities in patients undergoing total knee arthroplasty (TKA) is important for improving outcomes and resource utilization. Language barriers can adversely affect outcomes, though it is not clear whether the source of the disparity is language or a wider issue of racial, ethnic, or socioeconomic disparity. The purpose of this study was to compare short-term outcomes between English-speaking and non-English-speaking patients undergoing TKA.

Methods

A retrospective study of patients undergoing primary TKA within a single health system between January 2020 and December 2025 was performed. Non-English-speaking patients were propensity score matched 1:1 to English-speaking patients. After excluding patients lost to follow-up, 147 non-English-speaking and 140 English-speaking patients remained. Demographics, social vulnerability, clinical and patient-reported outcomes, and follow-up patterns were compared between groups.

Results

Non-English-speaking patients experienced clinical outcomes comparable to those of English-speaking patients across perioperative, 30-day, and one-year measures. There were no differences in response rates or scores on Patient-reported outcome measures (PROMs) between groups. Non-English-speaking patients generated significantly fewer patient-initiated MyChart (Epic Systems Corporation, Verona, Wisconsin, USA) electronic messages or telephone calls compared with English-speaking patients (1.6 ± 2.3 vs. 2.5 ± 4.1, p = 0.028). Additionally, non-English-speaking patients were significantly more likely to receive home physical therapy (20 (13.6%) vs. 5 (3.6%), p = 0.005).

Conclusion

Overall, non-English-speaking patients undergoing primary TKA achieved postoperative outcomes comparable to those of English-speaking patients despite demonstrating significantly greater social vulnerability. Additional studies are needed to replicate these results in a larger population and to identify any interventions that may prove beneficial in this population.

## Introduction

Total knee arthroplasty (TKA) is one of the most commonly performed orthopedic surgeries in the United States, and surgical volume is projected to continue to increase over time [1]. TKA is typically a successful procedure that results in decreased pain and improved quality of life for the patient and may reduce the need for healthcare resources over the long term [2,3]. Significant efforts have been devoted to identifying patient and system factors that contribute to complications and delayed recovery in order to improve outcomes for patients and decrease the stress on the healthcare system overall as volume increases [4-7].

Social factors, including minority status and socioeconomic disparities, have been shown in multiple studies to contribute to poorer outcomes in patients undergoing TKA [8-10]. Specifically, language barriers between patients and providers have been shown to adversely affect outcomes in some aspects of care among patients undergoing orthopedic procedures, including an increased risk of non-home discharge and longer length of stay, as well as increased hospital costs and reduced completion of patient-reported outcome measures (PROMs) [11]. Identifying existing disparities is vital in order to develop methods to address them and improve care for patients undergoing these procedures.

The purpose of this study is to compare short-term clinical and patient-reported outcomes, as well as postoperative follow-up engagement, between English-speaking and non-English-speaking patients undergoing primary TKA. We hypothesize that non-English-speaking patients will have worse overall clinical and patient-reported outcomes and reduced postoperative engagement compared with English-speaking patients.

## Materials and methods

Study design and patient selection

A retrospective observational study of patients undergoing primary, unilateral TKA at two affiliated acute-care hospitals and one affiliated ambulatory surgery center between January 2020 and December 2025 was performed. All surgeries were performed by board-certified orthopedic surgeons. All patients were cared for in a specialized joint replacement center with coordinated pre- and postoperative education, physical therapy, and nurse navigation services. Patients were classified as English-speaking or non-English-speaking based on the preferred language documented in the medical record. A total of 158 non-English-speaking patients were propensity score matched 1:1 to English-speaking patients. Covariates included in the propensity score model were age, sex, BMI, American Society of Anesthesiologists (ASA) physical status classification, Charlson Comorbidity Index (CCI), and insurance type. Patients with no postoperative follow-up were excluded post-matching. Complete loss-to-follow-up rates were 11.4% (18 patients) among English-speaking patients and 7.0% (11 patients) among non-English-speaking patients (p = 0.242). The final matched cohort comprised 147 non-English-speaking patients and 140 English-speaking patients.

Independent variables

Baseline demographic and clinical data collected included age, sex, BMI, race, ethnicity, insurance type, ASA score, CCI, and individual comorbidities (chronic pulmonary disease, congestive heart failure, diabetes, renal disease, and smoking status). Social vulnerability was quantified using the 2022 CDC Social Vulnerability Index (SVI), which encompasses four composite domains: socioeconomic status, household characteristics, racial and ethnic minority status, and housing type and transportation [12]. SVI scores range from zero to one, with higher scores indicating greater vulnerability.

Clinical outcomes

For non-English-speaking patients, primary language and interpreter utilization were recorded. Postoperative data collection included length of stay, discharge disposition, 30-day ED visits, and 30-day readmissions. One-year outcomes included medical complications (deep vein thrombosis/pulmonary embolism (DVT/PE), respiratory complications, and stroke), surgical complications (manipulation under anesthesia (MUA) and wound complications), reoperation, and opioid use. Opioid use was defined as still receiving opioid prescriptions from the surgical team that performed the primary surgery.

Follow-up and patient-reported outcomes

Postoperative follow-up engagement was assessed by the total number of scheduled follow-up appointments, missed or cancelled appointments, patient-initiated MyChart (Epic Systems Corporation, Verona, Wisconsin, USA) electronic messages or telephone calls, utilization of outpatient physical therapy, and utilization of home physical therapy.

PROMs were collected using the Patient-Reported Outcomes Measurement Information System (PROMIS) Physical Function (PF) short form, administered at baseline (preoperatively) and at one year postoperatively. The minimal clinically important difference (MCID) on the PROMIS-PF instrument was defined as an improvement of five points (1/2 standard deviation) from baseline. Patients who completed both baseline and one-year PF assessments were included in the MCID analyses.

Statistical analysis

Continuous variables are presented as mean ± SD and were compared between groups using independent-samples t-tests. Categorical variables are presented as frequency and percentage and were compared using chi-square tests or Fisher's exact test, as appropriate. Statistical significance was assessed at p < 0.05. All statistical analyses were performed using RStudio Version 1.4.171 (Posit Software, PBC, Boston, MA).

## Results

Patient demographics and social vulnerability

Following propensity score matching, non-English-speaking and English-speaking patients were well matched with respect to age (65.2 ± 9.1 vs. 65.5 ± 9.7 years, p=0.762), sex distribution (52 (35.4%) vs. 44 (31.4%) male, p=0.560), and BMI (32.9 ± 4.7 vs. 32.1 ± 5.8 kg/m², p=0.167). Despite matching, the cohorts differed significantly in racial composition and ethnicity, with non-English speakers more likely to identify as racial minorities (115 (78.2%) vs. 63 (45.0%) of a race other than White or Black/African American, p<0.001) and as Hispanic or Latino (118 (80.3%) vs. 82 (58.6%), p<0.001; Table 1).

**Table 1:** Demographics and comorbidities after propensity score matching. Data are expressed as mean ± SD or n (%). P-values <0.05 are shown in bold. χ² indicates the chi-square test of independence; t indicates the two-sample independent t-test; * indicates Fisher’s exact test. ASA: American Society of Anesthesiologists; CCI: Charlson Comorbidity Index; CHF: Congestive heart failure.

Demographics and comorbidities	English speakers (n=140)	Non-English speakers (n=147)	Test statistic	P-value
Age, years	65.5 ± 9.7	65.2 ± 9.1	t = 0.27	0.762
Sex				
Male	44 (31.4)	52 (35.4)	χ² = 0.34	0.56
Female	96 (68.6)	95 (64.6)		
BMI	32.1 ± 5.8	32.9 ± 4.7	t = -1.28	0.167
Race				
White	59 (42.1)	27 (18.4)	χ² = 34.30	<0.001
Black	18 (12.9)	5 (3.4)		
Other	63 (45.0)	115 (78.2)		
Ethnicity				
Hispanic or Latino	82 (58.6)	118 (80.3)	χ² = 15.99	<0.001
Not Hispanic or Latino	58 (41.4)	29 (19.7)		
Overall social vulnerability	0.58 ± 0.26	0.71 ± 0.28	t = -4.07	<0.001
Socioeconomic status	0.44 ± 0.30	0.61 ± 0.34	t = -4.48	<0.001
Household characteristics	0.57 ± 0.21	0.62 ± 0.20	t = -2.07	0.045
Racial and ethnic minority status	0.79 ± 0.19	0.86 ± 0.15	t = -3.47	0.001
Housing type and transportation	0.62 ± 0.22	0.73 ± 0.23	t = -4.14	<0.001
Medicaid insurance	27 (19.3)	16 (10.9)	χ² = 3.34	0.068
Medicare insurance	37 (26.4)	41 (27.9)	χ² = 0.02	0.884
Commercial insurance	59 (42.1)	81 (55.1)	χ² = 4.32	0.038
Workers’ compensation insurance	1 (0.7)	0 (0)	N/A	0.488*
Self-pay	1 (0.7)	5 (3.4)	N/A	0.215*
Unknown insurance	15 (10.7)	4 (2.7)	N/A	0.008*
ASA score ≥3	53 (37.9)	64 (43.5)	χ² = 0.74	0.391
CCI score	2.9 ± 1.5	2.9 ± 1.5	t = 0.12	0.903
Chronic pulmonary disease	26 (18.6)	17 (11.6)	χ² = 2.24	0.134
CHF	7 (5.0)	5 (3.4)	χ² = 0.15	0.703
Diabetes	22 (15.7)	35 (23.8)	χ² = 2.47	0.116
Renal disease	12 (8.6)	18 (12.2)	χ² = 0.68	0.41
Current smoker	4 (2.9)	4 (2.7)	N/A	1*
Former smoker	35 (25.0)	29 (19.7)	χ² = 0.85	0.352
Never smoked	101 (72.1)	114 (77.6)	χ² = 0.85	0.358
Chronic opioid use	1 (0.7)	1 (0.7)	N/A	1*

Non-English-speaking patients demonstrated significantly greater social vulnerability across all SVI domains compared with English speakers. Overall SVI scores were higher in the non-English-speaking cohort (0.71 ± 0.28 vs. 0.58 ± 0.26, p<0.001), with statistically significant differences observed in the socioeconomic status (0.61 ± 0.34 vs. 0.44 ± 0.30, p<0.001), household characteristics (0.62 ± 0.20 vs. 0.57 ± 0.21, p=0.045), racial and ethnic minority status (0.86 ± 0.15 vs. 0.79 ± 0.19, p=0.001), and housing type and transportation (0.73 ± 0.23 vs. 0.62 ± 0.22, p<0.001) domains. Non-English speakers were more likely to carry commercial insurance (81 (55.1%) vs. 59 (42.1%), p=0.038). Comorbidity burden, as measured by ASA score and CCI, was comparable between groups (p=0.391 and p=0.903, respectively), with no significant differences in individual comorbidities, including diabetes, renal disease, chronic pulmonary disease, congestive heart failure, or smoking status (all p>0.05).

Non-English speaker language characteristics

Among the 147 non-English-speaking patients, Spanish was the predominant primary language, accounting for 122 patients (83.0%). The remaining patients spoke a variety of languages, including Sign Language (n=5, 3.4%), Arabic (n=4, 2.7%), Russian (n=3, 2.0%), Dari (n=2, 1.4%), Gujarati (n=2, 1.4%), and Urdu (n=2, 1.4%), with one patient each speaking Albanian, Amharic, Finnish, French, German, Igbo, and Tigrinya. Professional interpreter services were required for 125 patients (85.0%; Table 2).

**Table 2:** Non-English-speaking patient details. Data are expressed as n (%).

Language	Non-English-speaking patients (n=147)
Albanian	1 (0.7)
Amharic	1 (0.7)
Arabic	4 (2.7)
Dari	2 (1.4)
Finnish	1 (0.7)
French	1 (0.7)
German	1 (0.7)
Gujarati	2 (1.4)
Igbo	1 (0.7)
Russian	3 (2.0)
Sign Language	5 (3.4)
Spanish	122 (83.0)
Tigrinya	1 (0.7)
Urdu	2 (1.4)
Interpreter needed	125 (85.0)

Clinical, 30-day, and one-year outcomes

Perioperative and postoperative outcomes were similar between the two cohorts (Table 3). Average length of stay was comparable between English- and non-English-speaking patients (1.1 ± 1.3 vs. 1.0 ± 1.4 days, p=0.716), and rates of non-home discharge did not differ significantly (9 (6.4%) vs. 6 (4.1%), p=0.530). At 30 days, rates of ED visits (9 (6.4%) vs. 7 (4.8%), p=0.721) and hospital readmission (2 (1.4%) vs. 2 (1.4%), p=1.000) were similar between groups.

**Table 3:** Clinical outcomes. Data are expressed as mean ± SD or n (%). P-values <0.05 are shown in bold. χ² indicates the chi-square test of independence; t indicates the two-sample independent t-test; * indicates Fisher’s exact test. DVT/PE: Deep vein thrombosis/Pulmonary embolism; MUA: Manipulation under anesthesia; I&D: Incision and drainage.

Outcomes	English speakers (n=140)	Non-English speakers (n=147)	Test statistic	P-value
Length of stay, days	1.1 ± 1.3	1.0 ± 1.4	t = 0.36	0.716
Non-home discharge	9 (6.4)	6 (4.1)	χ² = 0.39	0.53
30-day outcomes				
30-day ED visit	9 (6.4)	7 (4.8)	χ² = 0.13	0.721
30-day readmission	2 (1.4)	2 (1.4)	N/A	1*
1-year medical complication	4 (2.9)	2 (1.4)	N/A	0.438*
Medical complication type			N/A	1*
DVT/PE	1 (0.7)	1 (0.7)		
Respiratory	1 (0.7)	0 (0)		
Stroke	2 (1.4)	1 (0.7)		
1-year surgical complication	8 (5.7)	9 (6.1)	χ² = 0.00	1
Surgical complication type			N/A	0.294*
MUA	7 (5.0)	5 (3.4)		
Wound complication	1 (0.7)	4 (2.7)		
1-year reoperation	3 (2.1)	3 (2.1)	N/A	1*
Reoperation type			N/A	1*
I&D	1 (0.7)	1 (0.7)		
Patellar tendon reconstruction	0 (0)	1 (0.7)		
Revision (all components)	2 (1.4)	1 (0.7)		
1-year postoperative opioid use	1 (0.7)	0 (0)	N/A	0.880*

At one year, medical complication rates (4 (2.9%) vs. 2 (1.4%), p=0.438), surgical complication rates (8 (5.7%) vs. 9 (6.1%), p=1.000), reoperation rates (3 (2.1%) vs. 3 (2.1%), p=1.000), and postoperative opioid use (1 (0.7%) vs. 0 (0%), p=0.880) did not differ significantly between English- and non-English-speaking patients. Specific complication subtypes, including MUA, wound complications, DVT/PE, stroke, and revision arthroplasty, were also similar between groups.

Patient follow-up and engagement

Overall follow-up patterns were largely similar between groups; however, notable differences were observed in the modality of care utilization (Table 4). The mean number of follow-up appointments (4.0 ± 2.2 vs. 3.8 ± 1.6, p=0.464) and missed or cancelled appointments (1.0 ± 1.3 vs. 1.1 ± 1.3, p=0.466) did not differ significantly between English- and non-English-speaking patients.

**Table 4:** Patient follow-up and postoperative engagement. Data are expressed as mean ± SD or n (%). P-values <0.05 are shown in bold. χ² indicates the chi-square test of independence; t indicates the two-sample independent t-test.

Outcome	English speakers (n=140)	Non-English speakers (n=147)	Test statistic	P-value
Number of follow-up appointments	4.0 ± 2.2	3.8 ± 1.6	t = 0.88	0.464
Missed or cancelled appointments	1.0 ± 1.3	1.1 ± 1.3	t = -0.65	0.466
MyChart messages/calls	2.5 ± 4.1	1.6 ± 2.3	t = 2.28	0.028
Outpatient physical therapy	123 (87.9)	118 (80.3)	χ² = 2.53	0.112
Home physical therapy	5 (3.6)	20 (13.6)	χ² = 7.86	0.005

Non-English-speaking patients generated significantly fewer patient-initiated MyChart electronic messages or telephone calls compared with English-speaking patients (1.6 ± 2.3 vs. 2.5 ± 4.1, p=0.028). Additionally, non-English-speaking patients were significantly more likely to receive home physical therapy (20 (13.6%) vs. 5 (3.6%), p=0.005), while rates of outpatient physical therapy utilization were similar between cohorts (118 (80.3%) vs. 123 (87.9%), p=0.112).

Patient-reported outcome measures

Completion rates for baseline and one-year PROMIS-PF assessments were similar between groups, with no statistically significant differences in the proportions of patients with available baseline PF scores (111 (75.5%) vs. 117 (83.6%), p=0.129), one-year PF scores (34 (23.1%) vs. 44 (31.4%), p=0.148), or paired assessments suitable for longitudinal analysis (26 (17.7%) vs. 37 (26.4%), p=0.166; Table 5). Among those with available data, baseline PROMIS-PF scores were similar (35.7 ± 7.3 vs. 35.5 ± 6.3, p=0.839), as were one-year PF scores (39.4 ± 9.6 vs. 40.4 ± 7.4, p=0.609). The mean improvement in physical function did not differ between non-English- and English-speaking patients (5.9 ± 12.6 vs. 5.1 ± 8.0, p=0.779), and rates of MCID achievement were comparable (12 (46.2%) vs. 18 (48.6%), p=1.000).

**Table 5:** Patient-reported outcome measures. Data are expressed as mean ± SD or n (%). P-values <0.05 are shown in bold. χ² indicates the chi-square test of independence; t indicates the two-sample independent t-test. MCID: Minimal clinically important difference; PF: Physical function.

Outcome	English speakers (n=140)	Non-English speakers (n=147)	Test statistic	P-value
Had baseline physical function	117 (83.6)	111 (75.5)	χ² = 2.38	0.129
Baseline physical function score	35.5 ± 6.3	35.7 ± 7.3	t = −0.22	0.839
Had 1-year physical function	44 (31.4)	34 (23.1)	χ² = 2.09	0.148
1-year physical function score	40.4 ± 7.4	39.4 ± 9.6	t = 0.50	0.609
Had both baseline and 1-year PF	37 (26.4)	26 (17.7)	χ² = 2.71	0.166
Change in physical function	5.1 ± 8.0	5.9 ± 12.6	t = −0.29	0.779
MCID achievement	18 (48.6)	12 (46.2)	χ² = 0.00	1

## Discussion

In this propensity score-matched study of patients undergoing primary TKA, non-English-speaking patients experienced clinical outcomes comparable to those of English-speaking patients across perioperative, 30-day, and 1-year measures. Rates of 30-day ED visits, readmissions, medical and surgical complications, reoperation, and postoperative opioid use did not differ significantly between groups. However, differences emerged in patterns of postoperative engagement, as non-English-speaking patients generated fewer patient-initiated MyChart messages or phone calls and were more likely to receive home physical therapy. Notably, these findings occurred despite significantly higher levels of social vulnerability among non-English-speaking patients. Together, these results suggest that while language barriers and social vulnerability may influence how patients engage with postoperative care, they do not necessarily translate into worse clinical outcomes in the setting of structured perioperative management.

Previous studies examining the impact of limited English proficiency on outcomes after total joint arthroplasty have reported mixed findings. Nguyen KH et al. found higher rates of thromboembolic complications and longer hospital stays among patients with limited English proficiency, while Manuel SP et al. reported longer hospital stays, greater hospitalization costs, and increased discharge to skilled nursing facilities without differences in 30-day readmissions [13,14]. In a study looking specifically at opioid-prescription patterns, patients with decreased English language proficiency were less likely to request additional opioid pain relievers after TKA [15]. In contrast, we found no differences in perioperative, 30-day, or 1-year clinical outcomes between English- and non-English-speaking patients, including complications, readmissions, reoperation, ED visits, and postoperative opioid use. Our findings add to the growing body of literature suggesting that the association between limited English proficiency and postoperative outcomes may vary across patient populations and healthcare settings.

Notably, non-English-speaking patients in our cohort demonstrated significantly greater social vulnerability across all SVI domains than English-speaking patients. Previous studies have demonstrated that greater social vulnerability is associated with worse outcomes following total joint arthroplasty [10,16,17]. Vartanian KB et al. reported that increased social vulnerability is associated with higher rates of postoperative complications, longer hospital stays, increased readmissions, and greater healthcare utilization across orthopedic surgery [18]. Similarly, Pearson ZC et al. found that patients with one or more documented social determinants of health experienced significantly higher rates of complications, prosthetic joint infection, revision surgery, and readmission following TKA [5]. Despite these previously reported associations, non-English-speaking patients in our cohort experienced postoperative outcomes comparable to those of English-speaking patients. These findings suggest that higher social vulnerability does not necessarily translate into worse postoperative outcomes following primary TKA.

Although clinical outcomes were comparable between groups, differences emerged in postoperative engagement. Non-English-speaking patients generated fewer patient-initiated MyChart messages and telephone calls and were more likely to receive home physical therapy than English-speaking patients. These findings are consistent with prior studies demonstrating that patients with limited English proficiency have lower digital health literacy and reduced use of patient portals and electronic communication. Philpot LM et al. found that patients with limited English proficiency experienced greater difficulty engaging with digital healthcare tools, resulting in lower utilization of online health services and secure messaging [19]. Additionally, Semere W et al. demonstrated that caregivers, most commonly adult children, frequently play a central role in communicating with healthcare teams and navigating post-hospital care for patients with limited English proficiency [20]. Although caregiver involvement was not directly evaluated in our study, these findings may help explain the lower frequency of patient-initiated communication observed in our cohort. These findings highlight the importance of recognizing differences in postoperative engagement when caring for patients with limited English proficiency and suggest that communication strategies may need to be tailored to better support this population.

This study has several limitations. Its retrospective design is inherently subject to the biases of observational research, and because it was conducted within a single health system, the findings may not be generalizable to all patient populations or practice settings. Although propensity score matching was used to balance baseline characteristics between groups, significant differences remained in race, ethnicity, and social vulnerability, which may have affected the results. Patients lost to follow-up were excluded after matching was performed, which may have introduced additional confounding into the results. In addition, residual confounding from unmeasured variables remains possible. Given the relatively small sample size and few complications overall, this study is likely underpowered to detect significant differences, and it is possible that the negative findings may be the result of a type II error and should therefore be interpreted with caution. Completion rates for patient-reported outcome measures were low, limiting our ability to comprehensively evaluate functional recovery. Finally, while interpreter need was documented, the frequency, timing, and quality of interpreter utilization throughout the perioperative period were not assessed and may have influenced postoperative communication and patient engagement.

## Conclusions

Overall, non-English-speaking patients undergoing primary TKA achieved postoperative outcomes that were not significantly different from those of English-speaking patients, despite demonstrating significantly greater social vulnerability. However, important differences in postoperative engagement were observed, suggesting that traditional clinical outcome measures may not fully capture the care experiences of patients with limited English proficiency. Future studies should further evaluate strategies to improve communication and postoperative engagement while maintaining equitable clinical outcomes in this vulnerable population.
